# Subcutaneous Bupivacaine Infiltration Is Not Effective to Support Control of Postoperative Pain in Paediatric Patients Undergoing Spinal Surgery

**DOI:** 10.3390/jcm10112407

**Published:** 2021-05-29

**Authors:** Anna Danielewicz, Marek Fatyga, Grzegorz Starobrat, Monika Różańska-Boczula, Magdalena Wójciak, Ireneusz Sowa, Sławomir Dresler, Michał Latalski

**Affiliations:** 1 Department of Paediatric Orthopaedics, Medical University of Lublin, 20-093 Lublin, Poland; 2 Childrens’ Ortopeadic Department, Childrens’ University Hospital in Lublin, 20-093 Lublin, Poland; marekfatyga@umlub.pl (M.F.); grzegorz.starobrat@umlub.pl (G.S.); 3 Department of Applied Mathematics and Computer Science, University of Life Sciences in Lublin, 20-033 Lublin, Poland; monika.boczula@up.lublin.pl; 4 Department of Analytical Chemistry, Medical University of Lublin, 20-093 Lublin, Poland; magdalena.wojciak@umlub.pl (M.W.); i.sowa@umlub.pl (I.S.); slawomir.dresler@umlub.pl (S.D.)

**Keywords:** scoliosis, bupivacaine, spinal deformity, pain management

## Abstract

Spinal deformity corrections in paediatric patients are long-lasting procedures involving damage to many tissues and long pain exposure; therefore, effective pain management after surgical treatment is an important issue. In this study, the effect of inclusion of local infiltration analgesia, as an integral part of the scheme in postoperative pain control, in children and adolescents, subjected to the spinal deformity correction procedure, was assessed. Thirty patients, aged 8 to 17 years, undergoing spinal deformity correction were divided into a study group, receiving a 0.25% bupivacaine solution before wound closure, and a control group (no local analgesic agent). Morphine, at the doses of 0.10 mg/kg of body weight, was administered to the patients when pain occurred. Pain scores, morphine administration, and bleeding were observed during 48 postoperative hours. The pain scores were slightly lower in a 0–4 h period in patients who received bupivacaine compared with those in the control group. However, no differences were observed in a longer period of time and in the total opioid consumption. Moreover, increasing bleeding was observed in the bupivacaine-treated patients (study group) vs. the control. Bupivacaine only modestly affects analgesia and, due to the increased bleeding observed, it should not to be part of pain control management in young patients after spinal deformity correction.

## 1. Introduction

Only 0.1%. of paediatric patients suffering from scoliosis need surgical treatment; however, posterior spinal deformity corrections are long-lasting procedures, which require an extensive surgical approach, involving damage to many tissues and long pain exposure. They also carry the risk of many problems and complications [1,2]. The emerging surgical techniques and new implants have not yet brought about considerable change in the concept of the procedure, and the exposure of a substantial part of the spine is necessary, which certainly contributes to post-operative pain [3,4]. Effective pain management after posterior spinal fusion, in paediatric patients, is an essential part of surgical treatment for increasing patient’s comfort, allowing quicker convalescence. It also decreases the risk of complications, such as perioperative myocardial ischaemia, respiratory complications, immunological disorders, and postoperative cognitive disorders [5,6]. It also has an economic aspect, as it reduces the time of hospitalisation. Appropriate analgesia in paediatric patients is a complex issue, and multiple strategies are applied to prevent, or reduce, postoperative pain. In ward round practices, pharmacological treatment, including opioids, is regarded as the most effective; however, according to the WHO recommendation, reduction in drug administration is recently encouraged, due to the risk of adverse effects in patients [7]. Therefore, multimodal analgesia, which combines analgesics with local infiltration analgesia (LIA), as integral parts of the scheme in postoperative pain control, seems to be a promising solution [5]. One of the local analgesic agents is bupivacaine, synthesised in 1957, by Ekenstam. It reduces pain and muscle tension, causes loss of proprioception and sensation of temperature and touch, and shows an anti-inflammatory effect. Thus, bupivacaine has broad applications, including sympathetic, epidural, subarachnoid, block, and infiltration anaesthesia [8,9]. It can be used as a 0.25–0.50% solution applied directly before wound closure or in the form of catheter infusion, which allows gradual release of medication to the wound [6,10]. For example, incorporation of bupivacaine to a postoperative procedure proved effective in knee-joint alloplasty [11,12,13,14]. To date, however, there have been no reports proving the effectiveness of such a method in the case of paediatric patients who have undergone extensive procedures of the spinal region. Therefore, the aim of this study was to assess the effect of a combination of local infiltration analgesia, using bupivacaine, with the administration of opioids in children and adolescents subjected to the spinal deformity correction procedure. Three aspects were taken into consideration: (1) consumption of opioids in the period of 0–6 h, 6–24 h, and 24–48 h after the surgery, (2) pain intensity assessed based on the Numerical Rating Scale, (3) the influence of analgesia on bleeding in the early postoperative period.

## 2. Materials and Methods

### 2.1. Selection of Patients

The prospective cohort study involved patients treated for spinal deformity in the Paediatric Orthopaedics Department at the Children’s University Hospital in Lublin (Lublin, Poland) in 2015. The study was approved by the Bioethics Committee (consent no. KE-0254/105/2015). The oral and written consent was obtained prior to the surgery from all participants and their parents. Patients that (1) had allergies to bupivacaine, acetaminophen, or opioid, (2) had coagulation disorders, (3) were not fully responsive for physical, intellectual, and emotional reasons were excluded from the study. In collaboration with an anaesthesiologist team, the extended pain management protocol in spine surgery has been studied. Finally, the study involved 30 patients, including 13 children who followed standard perioperative protocol (control group) and 17 children who followed modified pain management protocol. The majority of subjects were patients with idiopathic scoliosis (23 persons), Scheuermann’s disease (4 persons), and congenital scoliosis (3 patients). The average number of segments involved was 11 in the study group and 12 in the control group (*p* = 0.92). Before the surgery, the patients were classified as I–III groups, according to the American Society of Anaesthesiologists (ASA) categorisation. The data about the patients and surgery are provided in Table 1.

The peripheral blood-count parameters and the coagulation profile of the patients are shown in the Supplementary material (Table S1). No statistically significant differences between the control and the study group were noted, and all results were within the standard values.

### 2.2. Procedure

The surgeries were carried out by two high-volume, fellowship-trained, and board-certified orthopaedic surgeons. The detailed premedication is described in the Supplementary material. Besides the standard analgesic treatment, the wound edges in the study group patients were injected with 10 mL of a 0.25% bupivacaine solution during the wound closure procedure. The pain intensity was assessed based on the Numerical Rating Scale (NRS) every 2 h during the 0–20 h period after the surgery. In the post-surgery anaesthesia, acetaminophen (dose: 15 mg per kg of body weight) was administered every 4 h, and morphine (dose: 0.1 mg per kg of body weight) was administered when the pain reached the level of 4 (moderate pain) or more. None of the patients developed general symptoms of the toxic effects of bupivacaine.

### 2.3. Statistical Analysis

All statistical analyses were conducted with the use of Statistica 10.0 software (StatSoft, Poland). A Kruskal-Wallis multiple comparison test, a T-distribution test, a chi-squared test, Mann-Whitney test, and Yule’s Q measure were used to analyse the differences between the control and the study group. The statistical significance was set at α = 0.05.

## 3. Results

### 3.1. Analgesic Effect

**Figure 1:**
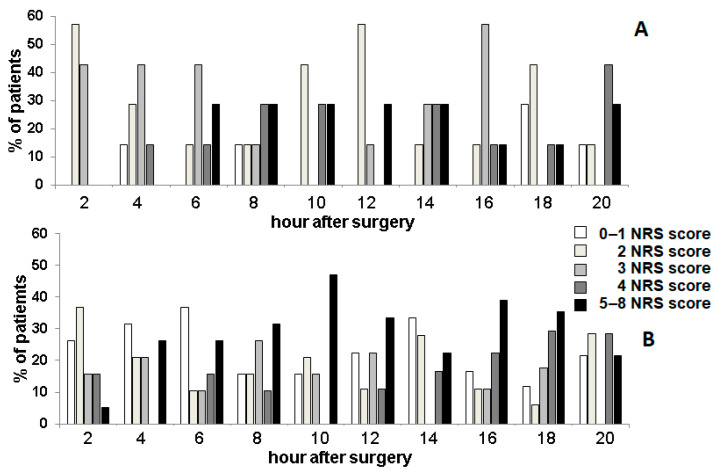
Assessment of pain according to the NRS scale in patients undergoing spinal deformity correction. (**A**)—study group, (**B**)—control group. In a longer time of observation, the pain scores were distributed to all five categories in both groups (data not shown).

The effect of bupivacaine on postoperative pain was investigated based on the pain scores according to the 10-point NRS scale, in which the child rates the pain from 0 (no pain) to 10 (worst imaginable pain). From 1 to 3, the pain was regarded as mild, and opioids were administered when the pain was described as 4 and above. The results were expressed as a percentage of patients who declared pain sensation at particular NRS values (Figure 1). 

As can be seen in Figure 1, the pain sensation was slightly lower in the study group; however, the effect was observed only up to 4 h after the surgery. No patients declared a pain score above 3 in the 0–2 h period, and only 14% of the patients described the pain as 4 in 2–4 h period. In contrast, 21% of patients in the control group declared pain above or equal to 4 in the period of 0–2 h, and 26% of the patients assessed the pain sensation as 5 in the period of 2–4 h. In a longer time of observation, the pain scores were distributed to all five categories in both groups.

**Figure 2:**
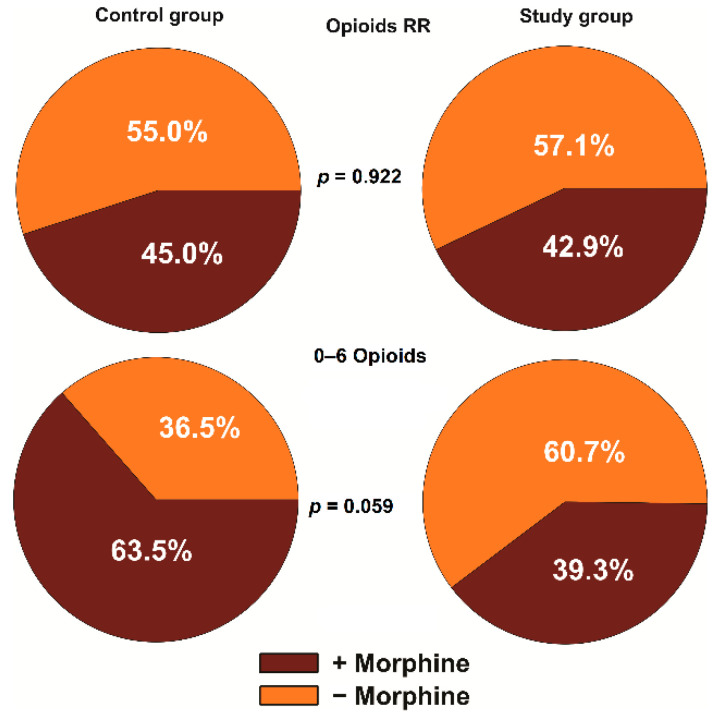
Percentage of patients who needed administration of morphine in the recovery room (RR) and within the 0–6 h period in the ward. The relationship between the control group and the study group were estimated using the chi-squared test.

The analgesic effect was also assessed based on the number of patients who needed opioid administration. The observations were conducted in the recovery room, and in the ward, in the period of 0–6 h, 6–24 h, and 24–48 h after the surgery. Generally, the chi-squared test did not show clearly significant differences between the patients in the control group and the study group within the tested periods. However, in the first 6 h in the ward, a slight effect of bupivacaine on the pain sensation was visible (Figure 2). In this period, 62.5% of patients required opioids in the control group vs. 39.3% of patients in the study group, and the estimated p value (*p* = 0.059) was close to the critical point of 0.05. No statistically significant differences were noted in the other periods of observation.

The total consumption of opiates in both groups (Figure S1) did not differ in a statistically significant manner. There were also no statistically significant differences in the time between the last dose of morphine given in the operating theatre and the first morphine administration in the ward, which was 403 and 362 min for the study and control groups, respectively (*p* = 0.54).

### 3.2. Effect on Bleeding

**Figure 3:**
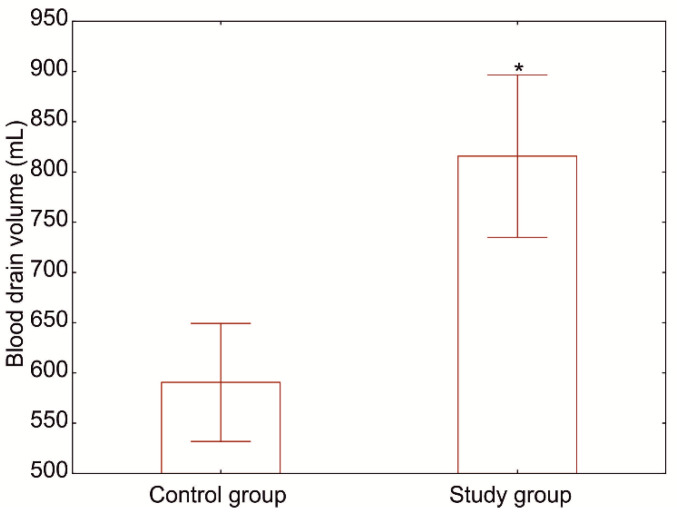
Blood loss during the postoperative period in the study and control groups (*p* = 0.0299).

The amount of blood in the drain and the time after which it was removed were also assessed. The average blood loss in the study group was 815 mL and 590 mL in the control group (Figure 3). The values differed in a statistically significant manner (*p* = 0.0299). The increased bleeding was not reflected in the frequency of packed red-blood-cell transfusion in the postoperative course, amounting to 30% and 35% in the study and control groups, respectively (*p* = 0.1766). 

No significant differences in the wound drainage time (*p* = 0.1235) and no correlations between the number of fixed segments and increased bleeding (*p* = 0.35) were observed.

## 4. Discussion

Local injections of postoperative wounds, as part of postoperative pain management, have proven to be effective in some orthopaedic, neurosurgical, and cardiothoracic surgical procedures [15]. The benefits of the intra-articular and periarticular administration of bupivacaine are widely described, especially in relation to patients after alloplasties and procedures performed on the large joints of the lower limbs [12,13,14]. It was shown that it reduced opiate doses administered, in the first 48 h after the surgery, and prolonged the time to the first administration of narcotic pain medications. The positive impact of bupivacaine wound injections on lung function after surgical procedures, in the abdominal area, was described in literature as well [16]. Jellish et al. and Waite et al. [17,18] pointed to the fact that wounds that have been injected or sprayed with an analgesic drug exhibited no interruptions to the wound healing process. 

However, some researchers do not observe the expected beneficial effects of LIA. As reported by Zmora et al., the intraperitoneal administration of bupivacaine does not lead to pain alleviation after laparoscopic cholecystectomy [19]. The results of tests among children and adolescents are also inconclusive [20]. Srinivasan et al. did not observe any differences between LIA and the single intrathecal administration of opioids to children subjected to Minimally Invasive Urologic Surgery, stating only that the former method carries a lower risk of complications [21]. In turn, a paper on the infiltration of postoperative wounds in children with cerebral palsy, after reconstruction within hip joints, has shown that epidural analgesia appears to be a far more effective method of pain management than LIA [22]. Similarly, Mattila et al. did not see any benefits of infiltration analgesia in children who underwent sternotomy due to atrial septal defect [23]. The two papers might indicate the insufficient efficacy of LIA in the case of surgeries that lead not only to damaged tissues but also to bone continuity disruptions.

There are not many papers presenting the use of LIA with regards to spinal surgeries, or they usually involve adult patients that are most frequently subjected to less extensive surgeries. Greze et al. evaluated the impact of ropivacaine, administered through a catheter, on pain experienced by patients undergoing posterior fixation, which included various numbers of segments. The authors did not obtain any satisfactory analgesic effects of LIA [24]. In turn, Puffer et al. assessed the effects of liposomal bupivacaine administered to microdiscectomy patients. In this case, the authors did not achieve any reduction in patients’ pain levels, according to the VAS. They also failed to reduce the total dose of opiate medications, shortening only the duration of the required administration [25]. In contrast, liposomal bupivacaine, injected into paraspinal musculature, demonstrated a reduced pain score in paediatric patients subjected to spinal surgery [26]. 

In the majority of papers, authors did not consider the impact of LIA on postoperative bleeding. Only Niemeläinen et al. mentioned this aspect of the performed procedures; however, in contrast to our findings, he did not observe increased blood loss after the surgery [12]. The increased bleeding may be reduced by the addition of adrenaline, i.e., a vasoconstrictor frequently used in local analgesia. For example, this solution was recommended by Chessman [27]; however, he did not provide any details of the amount, ratio, and effects of the medication. Other authors also confirm the beneficial analgesic and anti-bleeding effects of the inclusion of adrenaline to local analgesics [12,28].

The number of patients involved in our study was relatively low; however, an interim analysis revealed that bupivacaine infiltration was not effective to support control of postoperative pain in paediatric patients undergoing spinal surgery; thus, we decided to discontinue the study and not to extend the research with a larger group of patients.

## 5. Conclusions

This study aimed to assess the effectiveness of local injections of bupivacaine as part of postoperative analgesia in children undergoing surgical corrections due to spinal deformities. The investigation showed that the pain sensation was moderately reduced up to 4 h after the surgery in patients treated with bupivacaine; however, the study and control groups did not differ significantly in the time up to the first morphine administration in the ward and in the total amount of opiates administered during 48 h. Moreover, increased postoperative wound bleeding was observed in the study group. It may have been related to the fact that, besides their effect on fibres responsible for pain conductivity, the local anaesthetics led to the dilation of sympathetic fibres to skeletal vessels. Therefore, local infiltration analgesia with bupivacaine should not constitute a routine procedure in the group of young patients subjected to spinal deformity corrections. However, this observation did not exclude the effectiveness of LIA with bupivacaine in the case of surgeries with, potentially low, postoperative bleeding. Moreover, the addition of other analgesics and/or vasoconstrictors might increase the analgesic effects of treatment without an adverse effect on the amount of bleeding. Another idea worth considering, to improve postoperative pain control, can be Erector Spinae Plane Blockade. This could be a starting point for further studies.

## Figures and Tables

**Table 1:** Patients’ details.

	Study Group	Control Group
Number of patients (F/M)	17 (13/4)	13 (11/2)
Age	15.1 (12.6–17.5)	14.0 (8.5–17.0)
Weight (kg)	49.2 ± 9.3	49.5 ± 8.7
Duration of surgery (min.)	269.5 ± 33.2	280 ± 31.4
Number of segments	11 (10–11)	12 (11–12)

## Data Availability

The data presented in this study are available on request from the corresponding author.

## Supplementary Materials

The following are available online at https://www.mdpi.com/article/10.3390/jcm10112407/s1, Figure S1: Consumption of opiates in control and study group calculated on kg of body weight, Table S1: Red blood-cell parameters and coagulation in patients.
