# Supplementary material for: Subcutaneous Bupivacaine Infiltration Is Not Effective to Support Control of Postoperative Pain in Paediatric Patients Undergoing Spinal Surgery
Source: J Clin Med. 2021 May 29;10(11):2407. doi: 10.3390/jcm10112407 (PMC8198662; doi:10.3390/jcm10112407)
Supplement: Supplementary file 1 [file jcm-10-02407-s001.zip › jcm-1190054-supplementary.pdf]

Supplementary Material

Subcutaneous Bupivacaine infiltration is not effective to support control of postoperative pain in paediatric patients undergoing spinal surgery

Anna Danielewicz[1]*, Marek Fatyga[2], Grzegorz Starobrat[2], Monika Różańska-Boczula[3], Magdalena Wójciak[4], Ireneusz Sowa[4], Sławomir Dresler[4] and Michał Latalski[1]*

Premedication procedure

The patients received preoperatively a preventive course of antibiotics in accordance with the procedure adopted by the Clinic (Biofuroksym 100 mg per kg of body weight in three equally divided doses). The premedication in the operating theatre included the intravenous administration of midazolam (0.05-0.1 mg per kg of body weight up to a maximum of 3 mg) and acetaminophen (15 mg per kg of body weight). During the administration of anaesthesia the patients were given atropine (0.01 mg per kg of body weight up to a maximum of 0.5 mg), thiopental (the intravenous administration of 5 mg per kg of body weight), sufentanil (the intravenous administration of 1 μg/kg then an infusion of 1 μg/kg/h) or fentanyl (initial dose 2.5 μg/kg then 2-2.5 μg/kg as bolus doses every 30 minutes) and rocuronium (0.6 mg per kg of body weight). The breathing mixture used to support the anaesthesia contained 40% oxygen, 60% nitrous oxide and 2-3% sevoflurane.

Table S1. Red blood-cell parameters and coagulation in patients.

Parameters	Study group	Control group	Standard	P value*
RBC (10^6/uL)	4.83	4.79	M: 4.32–5.72<br>F: 4.1–5.3	0.8
Hb (g/dL)	14.2	13.95	M: 13.5–17.5<br>F: 12.2–14.8	0.99
Ht (%)	40.72	40.8	M: 38.8–50<br>F: 35.7–43	0.93
MCV (fL)	84.38	85.66	M: 81.2–95.1<br>F: 78.5–90.4	0.56
PLT (10^3/μL)	253	255	140–440	0.94
APTT (sec.)	31.6	31.95	25.8–38.6	0.59
PT (sec.)	12.4	12.35	13–17	0.68
PT-INR	1.1	1.085	0.8–1.2	0.85
Fibrinogen (g/L)	2.24	2.43	2.0–4.0	0.37

* Kruskal-Wallis multiple comparison test was used. The statistical significance was set at α=0.05.

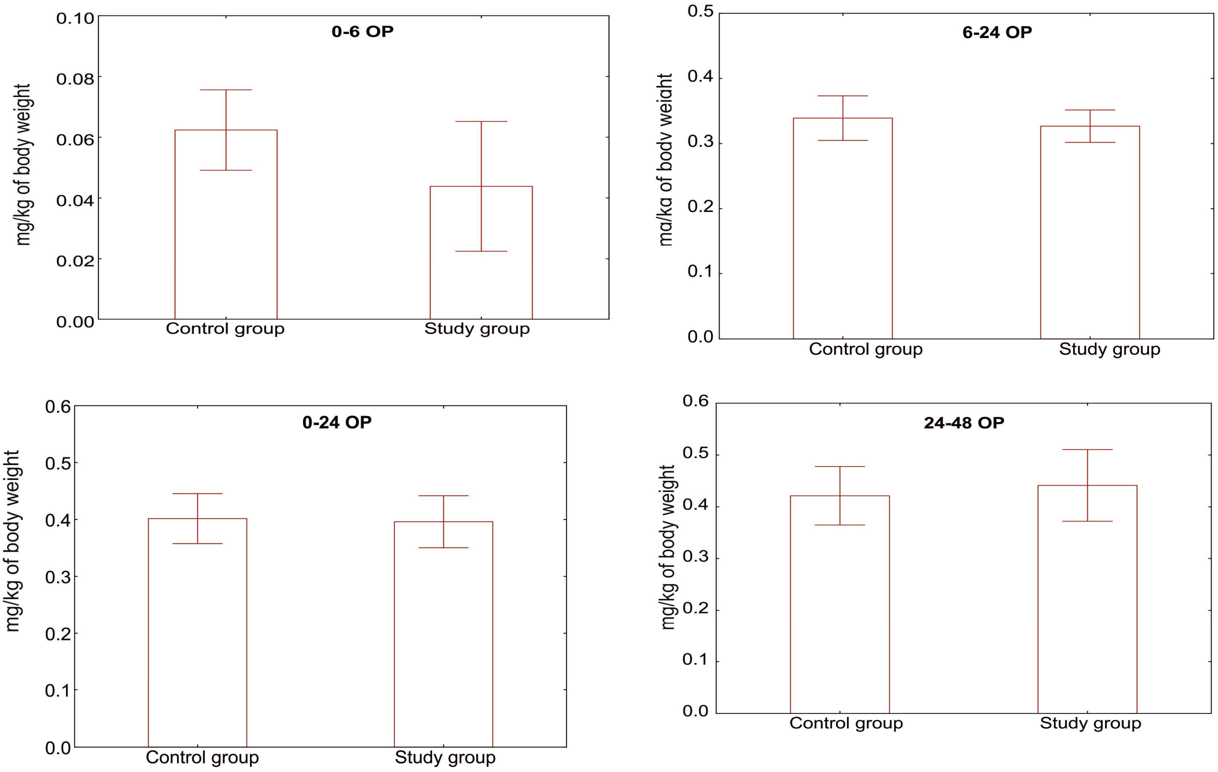

Figure S1. Consumption of opiates in control and study group calculated on kg of body weight.
